# Desenmascarando al imitador: neumonía por citomegalovirus que inicia como CVID se diagnostica como deficiencia de GATA2 con dos mutaciones patológicas únicas

**DOI:** 10.7705/biomedica.7813

**Published:** 2024-12-23

**Authors:** Andrés F Zea-Verano, Mónica Fernandes-Pineda

**Affiliations:** 1 Departamento de Microbiología, Facultad de Salud, Universidad del Valle, Cali, Colombia Universidad del Valle Universidad del Valle Cali Colombia; 2 Genetic Immunotherapy Section, Laboratory of Clinical Immunology and Microbiology, Division of Intramural Research, National Institute of Allergy and Infectious Diseases, National Institutes of Health, Bethesda, MD, USA National Institute of Allergy and Infectious Diseases National Institute of Allergy and Infectious Diseases USA; 3 Departamento de Medicina Interna, Universidad del Valle, Cali, Colombia Universidad del Valle Universidad del Valle Cali Colombia

**A la pregunta 1.** ¿Está de acuerdo con el diagnóstico inicial? ¿Cuáles serían los diagnósticos diferenciales?

Los diagnósticos diferenciales propuestos fueron:

1. Error innato de la inmunidad, deficiencia predominantemente de anticuerpos:

b. ¿Inmunodeficiencia común variable? 

c. ¿Agammaglobulinemia ligada al X, de aparición tardía? 

d. ¿Defectos de la Inmunidad intrínseca o innata? 

2. Neumonía recurrente secundaria a 1 

3. Enfermedad diseminada por citomegalovirus, resuelta 

4. Antecedente de neumonía no complicada por SARS-CoV-2 

5. Infecciones crónicas y otras causas de inmunodeficiencia secundaria, descartadas 

**A la pregunta 2.** ¿Qué estudios complementarios se solicitarían para este paciente?

Se consideró:

- Cuantificación de poblaciones linfocitarias extendidas; determinación de linfocitos T (CD4+ y CD8+), linfocitos B (CD19+/CD20+) y linfocitos NK (CD56)
- Subpoblaciones de linfocitos B para evaluar el cambio de isotipo y la presencia de linfocitos B de memoria
- Niveles valle de inmunoglobulina G (IgG)
- Secuenciación completa del exoma (WES) con énfasis en los genes relacionados con errores innatos de la inmunidad

## Seguimiento y resultados de los estudios complementarios

El paciente ha recibido de forma continua sustitución con inmunoglobulinas por vía intravenosa, con buena tolerancia. Durante la evolución, por razones administrativas, las valoraciones por inmunología no le fueron autorizadas y no pudo asistir a los controles.

Las poblaciones linfocitarias extendidas y las subpoblaciones B no se determinaron. Los niveles valle de IgG fueron de 5,16 g/L en mayo del 2023, tras 12 meses de sustitución con inmunoglobulinas por vía intravenosa.

El exoma reportó dos variantes: c.938A>G (p.His313Arg) y c.937C>T (p.His313Tyr) en el gen *GATA2*. Se desconoce si estas variantes están en *cis* o en *trans*; ambas variantes fueron categorizadas como variantes de significado clínico incierto, de acuerdo con los criterios del *American College of Medical Genetics* - ACMG ^1^. Sin embargo, se debe recalcar que estas variantes nunca han sido reportadas en la literatura o en las bases de datos (*Minor Allele Frequency*, MAF < 0,000001) y la predicción bioinformática para ambas variantes fue de patógena o deletérea (SIFT, MutationTaster, MutPred, PolyPhen-2) con un CADD de 33 (*Computer-Aided Drafting and Design*, CADD) que nos habla de su patogenicidad. La variante p.His313Tyr ya ha sido identificada en la deficiencia de *GATA2* como patológica y asociada con este error innato de la inmunidad ^2^.

El cuadro clínico, los hallazgos de laboratorio y el diagnóstico molecular, nos permitieron llegar al diagnóstico de deficiencia de *GATA2*. Este error innato de la inmunidad, también conocido como inmunodeficiencia 21 o MONOMAC, es una inmunodeficiencia primaria genética poco común, caracterizada disminución o ausencia de monocitos, células dendríticas circulantes y tisulares, linfocitos B y células asesinas, acompañada de propensión a infecciones por micobacterias, virus del papiloma y micosis, entre otras. Entre las manifestaciones clínicas más temidas, está el mayor riesgo de desarrollar neoplasias mieloides.

## Conclusión

Dados los hallazgos de una médula ósea con aumento de células y monocitos ligeramente aumentados durante la hospitalización (560 células/ μl en mayo del 2022), se decidió valorar la evolución de los monocitos y se encontraron 900 células/μl en mayo del 2023. Por todo esto, se encuentra en seguimiento y vigilancia por el gran riesgo de transformación a una leucemia mielomonocítica crónica dado su diagnóstico genético. Actualmente, está siendo sometido a los estudios haplogenéticos pertinentes, para considerar un trasplante de médula ósea como opción curativa.

La discusión de la segunda parte de este artículo la centraremos en tres aspectos que consideramos claves, a saber: 1) enfoque del paciente adulto con sospecha de un error innato de la inmunidad; 2) errores innatos de la inmunidad más frecuentemente encontrados en adultos, y 3) deficiencia de *GATA2*.

## Enfoque del adulto con sospecha de un error innato de la inmunidad

Los errores innatos de la inmunidad, tradicionalmente denominados inmunodeficiencias primarias, son un grupo heterogéneo de enfermedades monogénicas y fenocopias que afectan tanto a niños como a adultos ^3^. Las diferentes series estiman que entre el 30 y el 50 % de los casos de errores innatos de la inmunidad en todo el mundo, corresponden a personas mayores de edad ^4^.

En el enfoque del paciente con sospecha de un error innato de la inmunidad, la primera pregunta que nos debemos hacer es cuándo sospecharlo. A este respecto, algunas iniciativas -como la de la *Jeffrey Modell Foundation*- han trabajado en el establecimiento de los signos de alarma para un error innato de la inmunidad del adulto ^5^.

Los diez signos de alarma en el adulto son:

1. Dos o más infecciones de oído nuevas en un año;
2. Dos o más infecciones de senos paranasales en un año, en ausencia de alergia;
3. Una neumonía por año por más de un año;
4. Diarrea crónica con pérdida de peso;
5. Infecciones virales recurrentes (resfriados, herpes, verrugas, condilomas);
6. Necesidad recurrente de antibióticos endovenosos;
7. Abscesos recurrentes de la piel o de los órganos internos;
8. Moniliasis o infecciones micóticas de la piel o de otras localizaciones;
9. Infecciones con micobacterias, y
10. Historia familiar de un error innato de la inmunidad.

En nuestro medio, encontramos que los pacientes con un error innato de la inmunidad confirmado consultaron principalmente debido a infecciones recurrentes, un episodio de infección grave, infecciones oportunistas, síntomas de autoinflamación o autoinmunidad no claros o alteraciones en los resultados de laboratorio (hemograma) ^6^. No obstante, es importante destacar que hasta la tercera parte de los pacientes no cursaba con los signos clásicos de errores innatos de la inmunidad.

**Figura 1:**
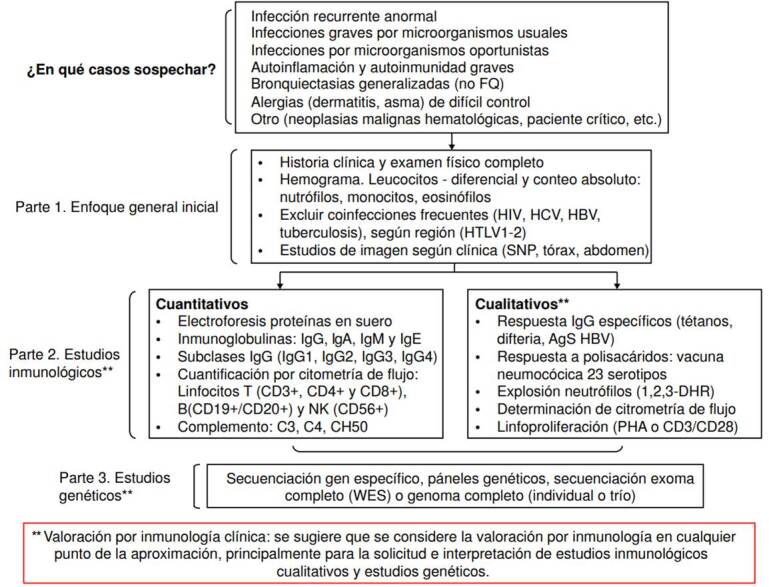
Algoritmo de evaluación inicial del paciente adulto con sospecha de error innato de la inmunidad

Teniendo en cuenta la literatura científica y la experiencia de más de 10 años del Servicio de Inmunología Clínica de la Universidad del Valle y el Hospital Universitario del Valle, proponemos un flujograma diagnóstico para los pacientes adultos con sospecha de un error innato de la inmunidad. Consideramos que, entre los signos de alarma de adultos, las bronquiectasias que no sean por fibrosis quística son un signo y una causa importantes de consulta en adultos ^7^ (figura 1), por lo cual esta debe tener una consideración especial adicional.

*AgS HBV: antígeno de superficie del virus de la hepatitis B; 1,2,3-DHR: dihidrorrodamina; FQ: fibrosis quística; HBV: virus de la hepatitis B; HCV: virus de la hepatitis C; HTLV1-2: virus linfotrópico de tipo 1 y 2 de las células T humanas; PHA: phytohemagglutinin; SPN: senos paranasales; HIV: virus de la inmunodeficiencia humana

## Errores innatos de la inmunidad más frecuentemente encontrados en adultos

La literatura científica no está completamente unificada respecto al punto de corte para considerar adultos a los pacientes (15, 18, 21 o 25 años) con errores innatos de la inmunidad, y esta falta de uniformidad tiene su principal sustento en tres hechos:

1. En muchos de los pacientes adultos con diagnóstico de un error innato de la inmunidad hecho durante la adultez, los síntomas se iniciaron en la niñez, lo cual significa, usualmente, un gran retraso diagnóstico ^6^;
2. Los pacientes con diagnóstico de un error innato de la inmunidad hacen la transición a la edad adulta y pasan de los servicios de pediatría, donde fueron diagnosticados y tratados por años, a los servicios de medicina interna ^8^, y
3. Están los pacientes cuyos síntomas y diagnóstico verdaderamente ocurren en la edad adulta.

De esta manera, sobre todo para los pacientes más jóvenes, es impreciso el momento en que se debe considerar como un error innato de la inmunidad de aparición en la edad adulta.

En forma global, podríamos afirmar que el grupo de las deficiencias predominantemente de anticuerpos -con la inmunodeficiencia común variable a la cabeza- representa cerca del 80 % de los pacientes adultos, diagnosticados en la edad adulta o en quienes se iniciaron síntomas pocos años antes de la adultez ^9^. Esto representa una gran ventaja, pero a la vez un gran reto. Si bien la mayoría de los pacientes con deficiencias de anticuerpos se pueden diagnosticar con estrategias relativamente sencillas en los estudios serológicos (determinación de inmunoglobulinas, subclases, anticuerpos específicos) y podrían beneficiarse de la sustitución de inmunoglobulinas, es también muy frecuente el no indagar más y quedarse satisfecho con un diagnóstico fenotípico sindrómico de hipogammaglobulinemia u otras deficiencias de anticuerpos, que puede estar enmascarando una etiología monogénica con consecuencias potencialmente más graves.

Los errores innatos de la inmunidad que clásicamente se presentan en la edad adulta son: el síndrome de Good, la linfopenia idiopática de CD4+, la deficiencia de *GATA2*, la inmunodeficiencia combinada de aparición tardía, la inmunodeficiencia común variable y la deficiencia selectiva de IgA ^10^, además de hipogammaglobulinemia de IgG o subclases en un grupo muy heterogéneo de sujetos. A pesar de ser estos los más frecuentes en la edad adulta, podemos afirmar que todos los grupos de errores innatos de la inmunidad presentes en la clasificación de la *International Union of Immunological Societies* (IUIS) pueden manifestarse por primera vez en la edad adulta ^11^.

Los mecanismos detrás del retraso en la aparición del cuadro clínico de un defecto que es congénito, ocurren por cambios epigenéticos, medioambientales, mutaciones hipomorfas y mutaciones somáticas ^12^; sin embargo, en la mayoría de los casos, no está claro por qué las manifestaciones clínicas se demoran tanto en el tiempo en presentarse.

El entendimiento de las bases moleculares de este grupo de errores innatos de la inmunidad nos ha permitido aprender que las mutaciones pueden producir efectos deletéreos parciales y dar origen a defectos hipomorfos que no se traducen en un cambio o en la pérdida completa de la función; esto, sumado a las mutaciones con aumento de la función -dominantes negativas, de penetrancia parcial, entre otras- hace que los posibles cuadros clínicos sean innumerables ^13^.

En la literatura científica hay varios artículos de revisión que presentan los errores innatos de la inmunidad de aparición en el adulto. No obstante, sugerimos dos, los cuales consideramos que presentan la información de una forma muy pedagógica: *Primary immune deficiencies in the adult: A previously underrecognized common condition*^14^ y *Monogenic adult-onset inborn errors of immunity*^12^.

## Deficiencia de *GATA2*

La deficiencia de *GATA2* fue recientemente descrita e integra diversas enfermedades y un amplio espectro clínico en un único diagnóstico genético. La anteriormente conocida como MonoMAC, o DCML (deficiencia de células dendríticas, monocitos, linfocitos B y células asesinas, entre otros), actualmente es categorizada dentro de los errores innatos de la inmunidad. Ocasionada por mutaciones heterocigotas en el gen *GATA2*, se manifiesta por la falta de un factor de transcripción clave de las células hematopoyéticas. Las alteraciones hematoinmunológicas se inician entre la segunda y la tercera década de la vida. Cursan con monocitopenia profunda, linfopenias y déficit de células B y células asesinas, junto con predisposición de los individuos al síndrome mielodisplásico y a la leucemia mieloide aguda ^15^.

El primer caso fue descrito en los Estados Unidos en 1989, y su diagnóstico genético se hizo después del 2011 ^16^. Actualmente, la Organización Mundial de la Salud (OMS) la considera parte de los síndromes de predisposición al cáncer (específicamente neoplasias mieloides), hereditaria y poco frecuente. Se asocia con variantes patógenas de la línea germinal de *GATA2* que pueden evolucionar a síndrome mielodisplásico o leucemia mieloide aguda, con potencial disfunción orgánica asociada ^17^.

Las manifestaciones clínicas tienen una considerable variabilidad, a menudo caracterizada por la propensión a las infecciones virales y fúngicas ^18^. Esto se evidencia por un patrón de infecciones recurrentes anormales, manifestadas por verrugas recurrentes causadas por el virus del papiloma humano (HPV), que son resistentes al tratamiento inicial; además, por infecciones fúngicas como aspergilosis, histoplasmosis y candidiasis, que se pueden desarrollar en forma muy grave o agresiva ^15^.

Entre otras manifestaciones sugestivas, se incluyen la sordera neurosensorial, la proteinosis alveolar pulmonar, las neoplasias hematológicas y la psoriasis, las cuales pueden iniciarse en la edad adulta y asociarse con hipogammaglobulinemia y con la reacción anormal de anticuerpos en los pacientes con infecciones recurrentes. Esto puede llevar a que se categorice inicialmente como una inmunodeficiencia variable común, sin pruebas genéticas ^19^^,^^20^.

El diagnóstico se enfoca desde el espectro inicial de manifestaciones clínicas ^21^. Los pacientes pueden presentar múltiples infecciones por virus, especialmente virus herpes (varicela zóster, virus de Epstein-Barr y citomegalovirus), agentes patógenos intracelulares y propensión a infecciones por micobacterias. Al igual que otros errores innatos de la inmunidad, se asocia con manifestaciones reumatológicas por desregulación inmunitaria (hiperlaxitud, osteoartritis, espondilitis anquilosante y artritis reumatoide seronegativa) ^22^.

En los cambios iniciales de los resultados de laboratorio, se encuentra neutropenia con monocitopenia grave ^23^. Sin embargo, en los casos de deficiencia de *GATA2* que presentan monocitosis, se debe sospechar estadios de leucemia premielomonocítica ^24^.

La deficiencia de *GATA2* se ha relacionado con neoplasias malignas hematológicas. Las mutaciones en la línea germinal de *GATA2* son necesarias, más no suficientes, para desarrollar neoplasias mieloides, dado que no todos los pacientes con deficiencia de *GATA2* evolucionan a neoplasias malignas hematológicas ^23^. El espectro del fenotipo de estos pacientes es amplio, y la penetrancia no está siempre dilucidada, lo que implica un reto diagnóstico ^19^. Sin embargo, las neoplasias malignas mieloides se presentan antes de los 40 años en el 80 % de los casos ^25^.

Al considerar los diagnósticos de exclusión para la deficiencia de *GATA2*, se deben tener en cuenta los siguientes diagnósticos diferenciales:

1. anemia de Diamond-Blackfan, que se asocia con la anemia diseritropoyética y la trombocitopenia ligada al cromosoma X;
2. infecciones virales recurrentes causadas por el virus del papiloma humano (HPV) con formación de verrugas;
3. propensión a las infecciones por micobacterias, como el déficit de IL-12, y
4. la menos frecuente agammaglobulinemia ligada al cromosoma X, que se diagnostica en la adultez y, generalmente, se asocia con manifestaciones clínicas leves o moderadas ^25^.

Respecto al tratamiento, el trasplante alogénico de médula ósea es el único tratamiento curativo para la deficiencia de *GATA2*, en el que se reversa el síndrome de insuficiencia medular ^26^. Sin embargo, dado el patrón de herencia autosómico dominante de esta enfermedad, se debe descartar el diagnóstico genético en los familiares al momento de considerar posibles donantes ^27^.

## Conclusión

Los adultos pueden cursar con errores innatos de la inmunidad que pueden parecer simples deficiencias de anticuerpos y que, en realidad, están enmascarando una enfermedad genética mucho más grave. La hipogammaglobulinemia observada en el presente paciente, se asocia con las anomalías en las células plasmáticas identificadas en individuos con mutaciones de *GATA2*. Esta correlación destaca el papel crítico de *GATA2* en sostener una población fuerte y saludable de células plasmáticas.

Nuestros hallazgos subrayan la imperiosa necesidad de practicar pruebas moleculares en los pacientes adultos, con el objetivo de excluir causas poco comunes como la deficiencia de *GATA2*, especialmente cuando hay anormalidades en las poblaciones linfocitarias y en los recuentos de monocitos.

Es importante ampliar nuestro panorama y aprovechar las herramientas moleculares, para llegar a una medicina de precisión, aun en la población adulta.
